# Protein–Ligand Interactions: Recent Advances in Biophysics, Biochemistry, and Bioinformatics

**DOI:** 10.3390/ijms26199576

**Published:** 2025-10-01

**Authors:** Igor A. Sedov, Yuriy F. Zuev

**Affiliations:** 1 Chemical Institute, Kazan Federal University, Kremlevskaya 18, Kazan 420008, Russia; 2 Kazan Institute of Biochemistry and Biophysics, FRC Kazan Scientific Center, Russian Academy of Sciences, Lobachevsky Str. 2/31, Kazan 420111, Russia

Protein–ligand interactions result in the formation of complexes between proteins and ligands, which can be small molecules or macromolecules including other proteins. In living organisms, protein–ligand interactions govern many important functions in biochemical processes, including but not limited to: enzyme catalysis involving chemical transformation of the enzyme-bound ligands, signal transduction through ligand (e.g., hormone) binding to receptors, gene regulation by transcription factors, and RNA-binding proteins that bind to specific DNA or RNA sequences [1].

Protein–ligand interactions are often viewed through the lens of the molecular recognition paradigm, referring to the high specificity and affinity of non-covalent binding observed for many proteins and their ligands. Our understanding of the mechanism of molecular recognition by proteins has moved from the lock-and-key principle proposed by Emil Fischer in 1894 [2] and induced-fit theory suggested in 1958 [3], which were based on the indirect evidence, to atomic-resolution experimental structures of numerous protein–ligand complexes [4] and the direct observation of binding events and conformational transitions accompanying them in molecular dynamics simulations [5,6,7]. A number of important peculiarities and features of protein–ligand interactions have been discovered, in particular:

− A conformational selection mechanism assuming that the crossing of free-energy barriers between protein conformations before the binding event [8,9] is likely to be at least as common as the induced fit mechanism, where the ligand binding induces a conformational change in the protein [10], and both mechanisms can be engaged in the same binding process [11,12].
− Weak and transient protein–ligand interactions characterized by low affinity constants and short lifetimes turn out to be widespread and biologically relevant [13,14].
− Allosteric binding, the interaction of a molecule with a protein at a site distinct from its active site, causing a conformational change or conformational redistribution that alters the enzyme or receptor activity, plays an important role in signaling and regulatory pathways, and enables the design of pharmacologically active allosteric modulators [15,16,17,18,19].
− Multivalent binding involves multivalent ligands simultaneously interacting with a number of receptors or a single receptor with multiple binding sites [20,21]. Multivalency is typical for antibody–antigen interactions, protein-polysaccharide binding, complexes of nucleic acids with DNA/RNA-binding proteins and transcription factors, and results in an enhanced affinity and sometimes selectivity, which can be utilized in the development of therapeutic agents [22,23,24,25].

The most significant challenges and advances in the field nowadays are linked to the demands of the pharmaceutical industry. The value of thermodynamic binding (affinity) constants is a fundamental property for drug design, which is usually improved during lead optimization to at least a nanomolar affinity. In addition to thermodynamic affinity, ligand binding/unbinding kinetics and drug–target residence time can influence the drug efficacy, and have also attracted the attention of researchers [26,27,28].

Experimental techniques to measure binding constants still suffer from methodological problems and are limited in accuracy [29,30,31], but the latter can be improved if a combination of methods is used [32]. Experimental binding constants for numerous protein–ligand systems are available from online databases like ChEMBL, BindingDB, and PubChem; however, they do not cover all literature data, and many of the results obtained in commercial studies remain unpublished. In contrast, 3D structures of virtually all studied protein–ligand complexes can be found in the Protein Data Bank (PDB) [4]. An increasing number of contributions to the PDB are obtained using the cryo-EM technique, offering high-resolution and avoiding the need for protein crystallization, which allows for the visualization of even previously inaccessible large molecular weight complexes in a near-native hydrated state [33].

In large-scale screening campaigns, high-throughput methods allowing for the detection of molecules without interfering optical or fluorescent labels are becoming increasingly popular. High-throughput mass spectrometry (HT-MS) [34,35] and high-throughput surface plasmon resonance (HT-SPR) [36,37] expand the breadth of targets for which the screening can be performed and enable direct probing of protein–ligand binding.

Prediction of the ligand binding pose and affinity using structural bioinformatics tools is extremely valuable in the early stages of drug design. The success of AlphaFold 2 [38] in predicting protein folds paved the way to the structure prediction of protein complexes using deep learning models. Recently released models, such as RosettaFold All-Atom [39], AlphaFold 3 [40], Chai-1 [41], and Boltz-1 [42], provide the 3D structure of different types of biomolecular assemblies by using only the primary structures of their constituents. Presently, these models and numerous deep-learning docking methods [43] do not always outperform conventional molecular docking approaches in the accuracy of the binding pose of small ligands to the defined binding site [43,44], but offer a huge development potential.

The accurate computational prediction of ligand affinity presents further challenges after correct pose prediction. Binding free energy calculations using rigorous statistical thermodynamic approaches reach relatively high accuracy, but are extremely time-consuming due to the need for exhaustive conformational sampling [45,46]. Much faster deep learning methods may soon achieve a comparable precision for small ligands [47,48]. At the same time, large-scale virtual screening campaigns can now use make-on-demand libraries containing billions of chemical compounds [49], which are ranked by rough docking scores or ligand similarity metrics rather than accurate binding free energy estimates. The alternatives to extensive library screening are fragment-based drug design in combination with evolutionary or Monte-Carlo optimization algorithms and developing deep-learning generative models outputting prospective ligands for the target protein [50]. The latter can be based on recurrent neural networks, variational autoencoders, generative adversarial networks, or diffusion models, and can be used to design small molecules, peptides, or proteins [51,52].

One very hot topic is the interaction of ligands with intrinsically disordered proteins (IDPs) lacking a fixed spatial structure, and intrinsically disordered regions of proteins (IDRs). IDPs and IDRs are involved in various diseases including presently incurable cancers and neurodegenerative disorders [53]. Hence, many of these represent attractive pharmacological targets, but usually interact with a low affinity, especially with small molecule ligands due to the absence of well-defined binding pockets [54,55]. A combination of experimental and computational approaches [56,57] is necessary to understand the influence of ligands on the broad conformational ensembles of IDPs and develop rational design approaches to discover promising binders [58,59,60].

All in all, the studies of protein–ligand interactions are of utmost importance in gaining deeper insights into crucial biological processes and developing novel pharmaceuticals. The papers in the present Special Issue [61,62,63,64,65] bring some further contributions to this enormous and rapidly growing field.
